# Using Twitter to Examine Smoking Behavior and Perceptions of Emerging Tobacco Products

**DOI:** 10.2196/jmir.2534

**Published:** 2013-08-29

**Authors:** Mark Myslín, Shu-Hong Zhu, Wendy Chapman, Mike Conway

**Affiliations:** ^1^Department of LinguisticsUniversity of California, San DiegoLa Jolla, CAUnited States; ^2^Department of Family and Preventive MedicineUniversity of California, San DiegoLa Jolla, CAUnited States; ^3^Department of MedicineUniversity of California, San DiegoLa Jolla, CAUnited States

**Keywords:** social media, twitter messaging, smoking, natural language processing

## Abstract

**Background:**

Social media platforms such as Twitter are rapidly becoming key resources for public health surveillance applications, yet little is known about Twitter users’ levels of informedness and sentiment toward tobacco, especially with regard to the emerging tobacco control challenges posed by hookah and electronic cigarettes.

**Objective:**

To develop a content and sentiment analysis of tobacco-related Twitter posts and build machine learning classifiers to detect tobacco-relevant posts and sentiment towards tobacco, with a particular focus on new and emerging products like hookah and electronic cigarettes.

**Methods:**

We collected 7362 tobacco-related Twitter posts at 15-day intervals from December 2011 to July 2012. Each tweet was manually classified using a triaxial scheme, capturing genre, theme, and sentiment. Using the collected data, machine-learning classifiers were trained to detect tobacco-related vs irrelevant tweets as well as positive vs negative sentiment, using Naïve Bayes, k-nearest neighbors, and Support Vector Machine (SVM) algorithms. Finally, phi contingency coefficients were computed between each of the categories to discover emergent patterns.

**Results:**

The most prevalent genres were first- and second-hand experience and opinion, and the most frequent themes were hookah, cessation, and pleasure. Sentiment toward tobacco was overall more positive (1939/4215, 46% of tweets) than negative (1349/4215, 32%) or neutral among tweets mentioning it, even excluding the 9% of tweets categorized as marketing. Three separate metrics converged to support an emergent distinction between, on one hand, hookah and electronic cigarettes corresponding to positive sentiment, and on the other hand, traditional tobacco products and more general references corresponding to negative sentiment. These metrics included correlations between categories in the annotation scheme (phi_*hookah-positive*_=0.39; phi_*e-cigs-positive*_=0.19); correlations between search keywords and sentiment (χ^2^_4_=414.50, *P*<.001, Cramer’s *V*=0.36), and the most discriminating unigram features for positive and negative sentiment ranked by log odds ratio in the machine learning component of the study. In the automated classification tasks, SVMs using a relatively small number of unigram features (500) achieved best performance in discriminating tobacco-related from unrelated tweets (*F* score=0.85).

**Conclusions:**

Novel insights available through Twitter for tobacco surveillance are attested through the high prevalence of positive sentiment. This positive sentiment is correlated in complex ways with social image, personal experience, and recently popular products such as hookah and electronic cigarettes. Several apparent perceptual disconnects between these products and their health effects suggest opportunities for tobacco control education. Finally, machine classification of tobacco-related posts shows a promising edge over strictly keyword-based approaches, yielding an improved signal-to-noise ratio in Twitter data and paving the way for automated tobacco surveillance applications.

## Introduction

### Background

Social media platforms such as Twitter are rapidly becoming key resources for public health surveillance applications. Vast amounts of freely available, user-generated online content, in addition to allowing for efficient and potentially automated, real-time monitoring of public sentiment and informedness, allow for bottom-up discovery of emergent patterns that may not be readily detectable using traditional surveillance methodologies such as pre-formulated surveys. In this study, we demonstrate the feasibility of a Twitter-based “infoveillance” [1] methodology to monitor perceptions of tobacco usage, with a special focus on new public health challenges posed by hookah and electronic cigarettes (e-cigarettes). In particular, we collected a large corpus of tobacco-related Twitter posts, developed a specialized content analysis of these posts, and trained machine-learning algorithms to classify posts automatically according to their relevance to tobacco-related content categories.

### Twitter and Public Health Surveillance

Twitter offers a number of key benefits as a data source for public health surveillance. First, the dataset is large and readily accessible. In 2012, 340 million tweets were being posted daily [2], and this content is freely available (albeit subject to legal restrictions on redistribution). Second, data may be automatically collected and analyzed in real time. Third, Twitter content is user-centric, thus reflecting trends that surveys may not capture or that users may not discuss in more formal contexts. Finally, Twitter demographics allow for greater representation of underserved and difficult-to-reach groups. African-American, Hispanic, younger, and urban populations are in fact overrepresented on Twitter relative to the general population [3]. Twitter use is most common among 18-29 (26% of whom use Twitter) and 30-49 year olds (14% of whom use Twitter). Men and women use Twitter in almost equal numbers. Twitter users reflect the general population in terms of education levels: 15% of US adults with Internet access use Twitter (8% on a typical day), and 9% of US adults use Twitter from their smartphones (and with the projected growth of smartphone use, this number is likely to increase) [4].

Recent applications have sought to harness the unique public health surveillance opportunities offered by Twitter. A number of studies have tracked public sentiment and informedness during natural disasters, such as the 2011 Tohoku earthquake [5] and various disease outbreaks [1,6-9]. High correlations are reported between Twitter statistics and Centers for Disease Control and Prevention (CDC) statistics with regard to influenza informedness, affirming the value of Twitter as a rapid, cost-effective health status surveillance methodology [10]. In a related vein, Twitter surveillance has revealed evidence of poor public informedness and misuse of antibiotics [11]. In addition to tracking public sentiment and information, Twitter has been used to monitor medical conditions, such as dental pain in the community [12]. Finally, temporal patterns, such as those in problem drinking [13] and seasonal mood variation [14], have also been demonstrated using Twitter surveillance.

### Twitter and Tobacco Surveillance

“Infoveillance” is defined by Eysenbach as “the science of distribution and determinants of information in an electronic medium, specifically the Internet, or in a population, with the ultimate aim to inform public health and public policy” [1]. We believe that a Twitter-based infoveillance methodology can be profitably implemented in tobacco surveillance. Tobacco control is identified as a global public health priority by the World Health Organization [15], and tobacco use is the most preventable cause of disease in the United States. 400,000 smokers and former smokers die each year of smoking-related diseases, with an additional 38,000 nonsmokers dying prematurely due to second-hand smoke [16,17]. In this work, we are focused on using social media to analyze public perceptions of new and emerging tobacco control challenges, specifically hookah and e-cigarettes.

Preliminary tobacco research using Twitter data has addressed several specific domains. Freeman [18] analyzed use of social media by tobacco companies to promote smoking, while Lowe et al and Prochaska et al [19,20] studied Twitter-based smoking cessation networks. Finally, Prier et al [21] identified and classified tweets using automatic topic modeling. In our research, we built on this work in three key ways: (1) we developed a comprehensive, multidimensional content analysis, (2) we improved the signal-to-noise ratio in tobacco-related Twitter data by implementing machine learning classification techniques, and (3) we additionally focused on the special utility of Twitter surveillance for two new tobacco-related public health challenges, namely hookah and e-cigarettes. Note that the United States Federal Drug Administration (USFDA) has recently prioritized research on investigating public perceptions of new and emerging tobacco products, like hookah and e-cigarettes [22].

### Objective

Our first objective was to provide a content analysis of tobacco-related tweets. Work reported by Prier et al [21] identifies major topic categories using Latent Dirichlet Allocation topic models [23]. The five emergent categories identified using the topic modeling methodology were tobacco abuse, tobacco cessation, promotion of bars and marijuana smoking, anti-smoking content, and a general, incohesive category. We built on this work by manually developing a comprehensive and multidimensional taxonomy of tobacco-related tweets that could then be employed in machine learning applications. Using the first four categories identified by Prier et al as a starting point, we used an iterative content analysis technique [24] to build a multidimensional annotation scheme of tobacco-related tweets reflecting those categories important for public health.

Our second objective was to improve the signal-to-noise ratio in Twitter data by automatically filtering out irrelevant content. Strictly keyword-based approaches are susceptible to lexical ambiguity in natural language: the keyword and wildcard combination smok*, for example, matches not only tobacco-related tweets but also tweets referring to *smoked cheese.* In order to reduce the presence of this type of noise, we trained machine classifiers to distinguish between tobacco-related and unrelated tweets.

The third distinctive objective of our work was to demonstrate the utility of Twitter in addressing new public health challenges related to tobacco usage. Two such issues are the growing popularity of hookah and e-cigarettes. As we discuss below, Twitter surveillance is particularly suited to understanding these new challenges.

A hookah (also known as shisha or narghile) is a waterpipe used to smoke flavored tobacco. Hookah is smoked by an estimated 100 million people daily [25], with increasing numbers of users both in the United States and worldwide, especially among college-age adults [26-28]. While the health risks associated with hookah use are similar to those of cigarette smoking [29], perceptions are widespread that hookah is safer [28,30]. Further, waterpipe usage is subject to fewer regulations than cigarettes, with frequent exemptions on bans in bars [31]. Furthermore, hookah products are easily accessible via Internet marketing sites and in venues that do not verify age [28]. Despite the growing list of health concerns associated with hookah, no interventions have been designed specifically for this form of tobacco use [32]. Its growing popularity among young users and its widespread availability online make Twitter a key resource for its surveillance.

E-cigarettes (or e-cigs) are another recently popular tobacco product subject to only sparse regulation and research. An e-cigarette is an electronic inhaler that produces vapor to simulate cigarette smoking and that may or may not contain nicotine. While e-cigarettes have surged in popularity as cessation devices, no consensus exists among public heath researchers regarding their health effects, and they are not endorsed by either the USFDA or the CDC [33]. Indeed, some researchers show that e-cigarettes carry health risks and could appeal to nonsmokers, especially children, due to their novelty, flavorings, and possibly overstated claims of safety [34].

Regulation of e-cigarettes is sparse and variable by jurisdiction—no warning labels are required, and the product is easily available online [35]. Indeed, online marketing has surged [20], and by September 2010, Google searches related to e-cigarettes were several-hundred-fold more frequent than those related to cessation medications [36]. The centrality of the Internet to the rise of e-cigarettes underscores the value of Twitter surveillance of this product.

## Methods

### Data Collection

Using the Twitter Application Programming Interface (API), we collected a sample of tweets between November 2011 and July 2012 that represented 1% of the entire Twitter feed. This 1% sample consisted of an average 1.3 million tweets per day. In order to extract tobacco-related tweets from this dataset, we constructed a list of keywords relevant to general tobacco usage as well as hookah and e-cigarettes. Our initial list consisted of 30 such terms culled from online slang dictionaries, but we pruned this list to the 11 terms that were attested more than once per day in our Twitter sample (see below). These were cig*, *nicotine*, smok*, *tobacco*; *hookah*, *shisha*, *waterpipe*; *e-juice*, *e-liquid*, *vape*, and *vaping* (where * is a wildcard such that cig* matches tweets containing *cigar*, *e-cig*, and so on).

Our initial dataset included all tweets containing these keywords at 15-day intervals from December 5, 2011, to July 17, 2012, inclusive, which results in equal sampling of each day of the week. We thus avoided potential bias based on day of the week, which has been observed for alcohol-related tweets, which spike in positive sentiment on Fridays and Saturdays [13]. For each of the 16 days resulting from our sampling technique, all tweets matching any of the listed keywords were included. Tweets matching these tobacco-related keywords reflected 0.17% of all tweets in the Twitter API 1% sample. The vast majority of these keyword-relevant tweets corresponded to unique Twitter users: on average, each username was associated with 1.07 tweets in the sample.

One of our keywords, smok*, was dramatically more frequent and ambiguous than any of the others, matching far more tobacco-irrelevant tweets (for example, tweets referring to *smoked cheese*). In a preliminary sample of 500 smok* tweets, only 16.8% were relevant to tobacco according to manual classification. Furthermore, over 100,000 smok* tweets were included in our 16-day dataset, making hand classification impractical. We thus included smok* tweets only for days where there were less than 400 total tweets matched by all other keywords, so that each day’s total tweet count was at least 400, ensuring a balance such that no individual date was underrepresented. Following this procedure, 0.04% of all smok* tweets were included in the dataset. The resulting final dataset thus contained 7362 tweets, with a mean of 460 tweets per day (SD 35).

### Manual Content and Sentiment Analysis

**Figure 1:**
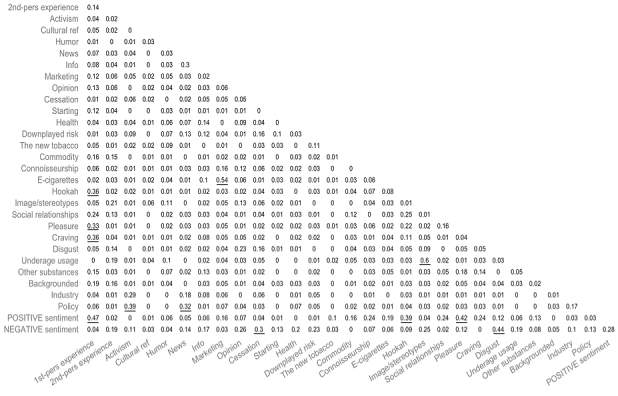
Correlations between all pairwise combinations of categories; values range from 0-1; correlations greater than 0.3 are underlined.

**Figure 2:**
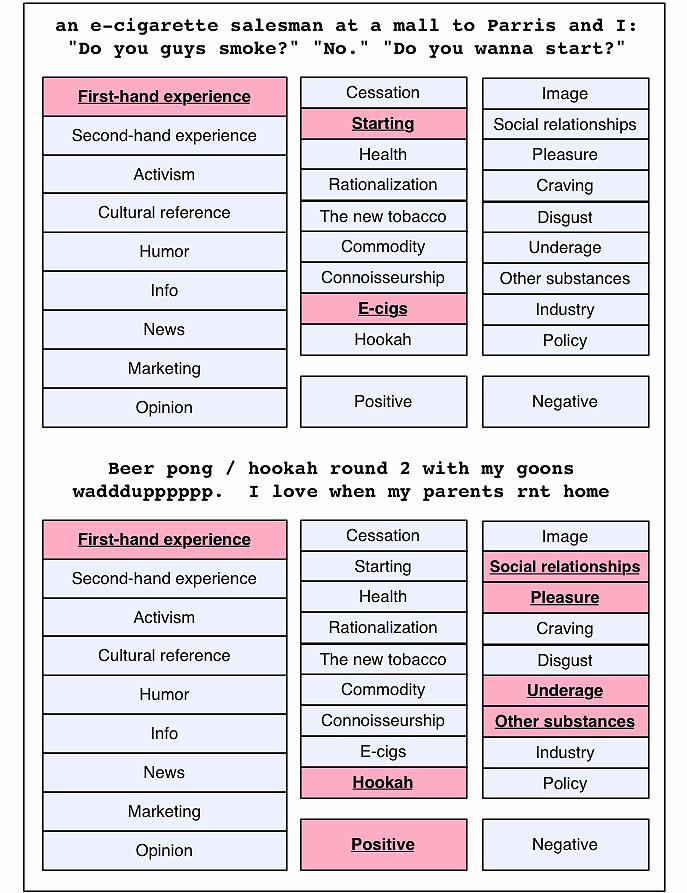
Example tweets manually classified using annotation scheme (relevant categories are shaded).

We developed a triaxial classification scheme to capture each tweet’s genre, theme, and sentiment. The former two axes are similar in scope to the content and qualifier categories developed in Chew & Eysenbach [7]. Genre reflects the format of the tweet (for example, formulaic joke, news item, or personal experience), and theme reflects the domain of the actual content conveyed (including such categories as health issues, underage usage, and tobacco policy). Sentiment, the third axis of the coding scheme, simply encodes the stance expressed in the tweet toward tobacco or its users, whether positive, negative, or neutral. Categories within each of the three axes were developed iteratively on the basis of a separate pilot dataset of approximately 1000 tweets from another date, which 2 annotators (authors MM and MC) classified according to an early version of the scheme. Upon review and discussion, several overly broad categories were split, while sparse, related categories were collapsed. A final version of the coding scheme was adopted when interannotator agreement among the 2 annotators on a set of 150 tweets exceeded a kappa level of 0.7 for each of the three axes. A complete list of all categories within this scheme is available in Figure 1, and detailed descriptions and example tweets for each category are presented in Multimedia Appendix 1. Figure 2 shows two examples of how tweets are classified using the annotation scheme.

The set of 7362 tweets was then manually classified according to the final version of this scheme by the 2 annotators. Tweets were assigned multiple categories within a single axis if applicable, and duplicate or re-tweeted posts were included only once to prevent spam or overly popular posts from biasing the sample. Non-English, unintelligible, or tobacco-irrelevant tweets were coded as belonging to none of the categories in the classification scheme.

### Intercategory Correlations

In order to discover emergent trends in tobacco-related Twitter content, we computed correlations for each pairwise combination of the 30 categories within the entire coding scheme. In other words, given two categories such as *hookah* and *positive sentiment*, we compared the number of tweets manually classified under both categories to the number expected by chance to be classified under both categories. The contingency coefficient phi (which is equivalent to Cramer’s *V* in the current 2×2 case) equals the square root of χ^2^/*n*, where χ^2^ is the chi-square statistic for the 2×2 contingency table, and *n* is the total number of observations. The phi coefficient ranges from 0 to 1, with 0 indicating no correlation between the two categories and 1 indicating perfect correlation.

### Machine Learning

We compared the performance of several machine learning algorithms on three classification tasks on the corpus of manually annotated tweets: relevance to tobacco, positive sentiment, and negative sentiment. Relevance to tobacco was operationalized as whether the tweet was classified under any of the categories in the scheme. Our goal was to test the feasibility of creating a natural language processing machine learning classifier with which we could automatically identify tobacco-related tweets in real-time.

**Figure 3:**
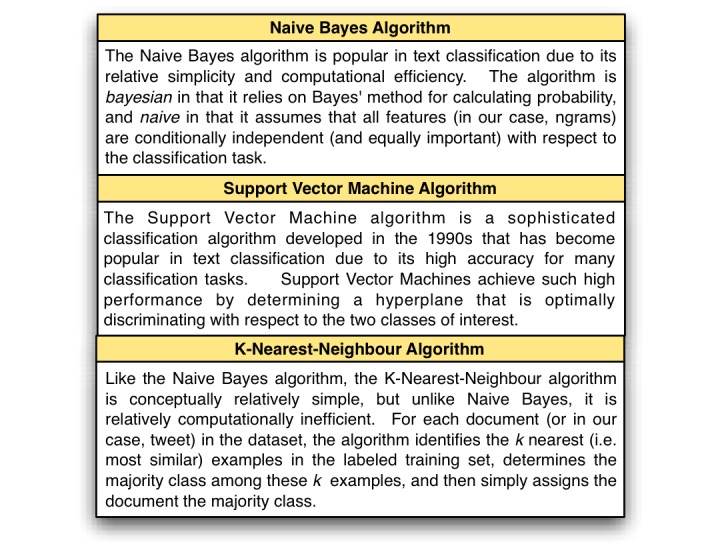
Machine learning algorithm description.

**Figure 4:**
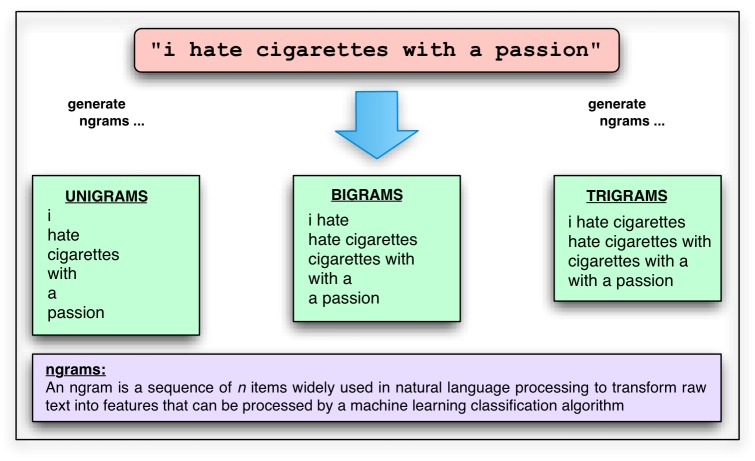
N-gram text representation.

We varied three parameters for each task: the machine learning algorithm, the order of n-gram used as features, and the number of features used. Algorithms used were Naïve Bayes, k-nearest-neighbors (KNN), and Support Vector Machines (SVM) (see Figure 3 for a brief description of these algorithms) [37], and features were either unigrams, bigrams, or trigrams (see Figure 4 for a description of n-grams). The number of features ranged from 1 to the number of unique n-grams present for the current task, tested at approximately logarithmic intervals from 1 to 1000 and at intervals of 500 thereafter. Feature selection was determined by information gain, which measures the increase in bits of information when a term is present versus absent (see, for example, Yang & Pedersen for discussion [38]). Comparative studies of feature selection metrics report information gain as one of the best-performing metrics for text classification [38,39]. Our goal in using feature selection was twofold. First, we wanted to identify those words and phrases most associated with tobacco-related tweets. Second, we wanted to use these high-quality features in order to increase the classification accuracy of our machine learning classifiers.

We employed the Rainbow toolkit [40] to train and test the above classifiers and manually implemented a 10-fold cross-validation routine for each classification task using the hand-annotated dataset. In cross-validation, the entire hand-annotated dataset is broken into *k* equally sized folds, and the classification task is performed *k* times, each time with a different fold held out as test data, and all other folds included as training data. Classification accuracies for each of the *k* iterations are then simply averaged.

**Figure 5:**
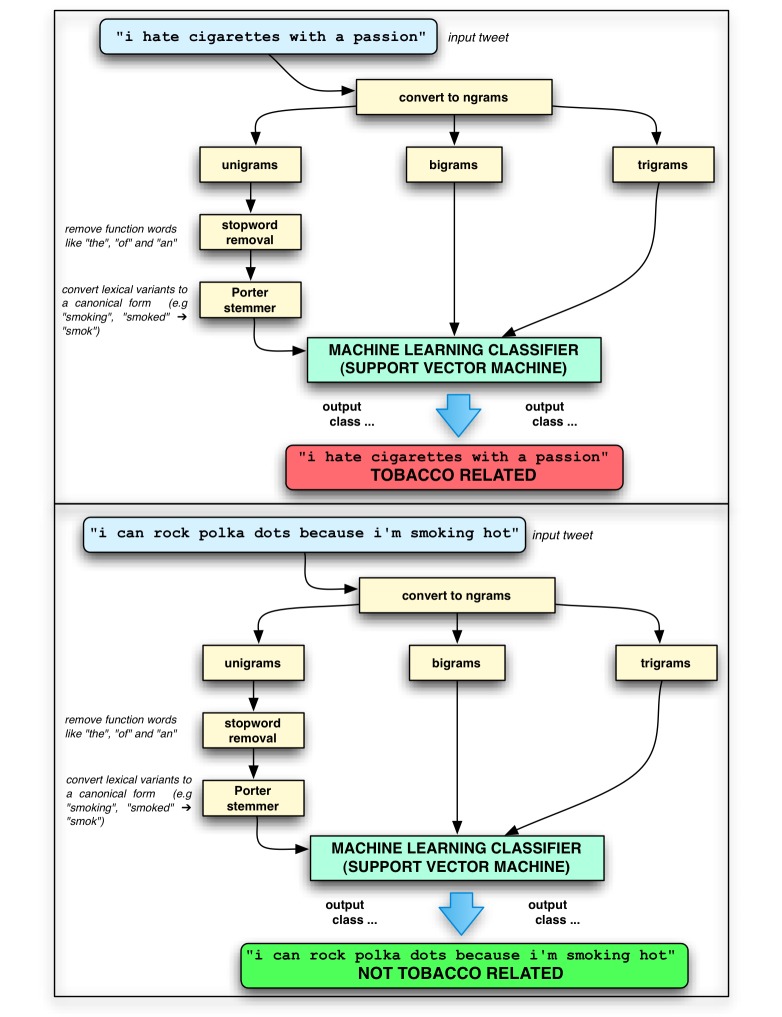
Machine learning experiment workflow.

Features used for machine learning were represented as binary presence/absence of words in a tweet rather than the number of times each term occurred in a tweet. Term frequencies are unlikely to be significantly more informative, since words are relatively rarely repeated within tweets (mean type-token ratio 0.96, SD 0.08). Two additional standard feature-processing measures were taken: first, all tweets were passed through the Porter stemmer [41], which converts words (such as *smoked* and *smoking*) to their bare stems (in both cases here, *smok*), so that different conjugations of the same lexical item are not counted as distinct features. Second, extremely frequent function words such as *the* and *is*, which are unlikely to be relevant to the classification task, were excluded as features using the standard 524-word SMART stoplist [42]. All other machine learning parameters were Rainbow defaults. Figure 5 summarizes the machine learning workflow.

In order to evaluate the machine learning results, five standard classification metrics were computed for each task. Accuracy is simply the percentage of tweets correctly classified by the algorithm. We also computed precision, recall, specificity, and *F* scores, which are defined in Multimedia Appendix 2.

## Results

### Content and Sentiment Analysis

The corpus of 7362 tweets was annotated by authors MM and MC according to the classification scheme described in the Methods section. Interannotator agreement (kappa) met the standard threshold of 0.7 for each of the three axes of the scheme: genre=0.78, theme=0.70, sentiment=0.77. Of the tweets, 4215 (57.3%) were classified as relevant to tobacco, with the remainder comprising tweets that were not in English or that matched alternate senses of their keyword, such as *smoked cheese* in the case of smok*.

Among the tobacco-related tweets (ie, 4215 out of a total of 7362), the most prevalent genre was first-hand experience, matching 40% of tweets, followed by second-hand experience (14%), and opinion (9%) (recall that tweets may be assigned multiple categories). The top themes were hookah (20%), cessation (14%), and pleasure (11%). Finally, sentiment toward tobacco was overall more positive (46% of tweets) than negative (32%) or neutral, even excluding the 9% of tweets categorized as marketing, which resulted in a 41%/30% positive/negative ratio.

**Figure 6:**
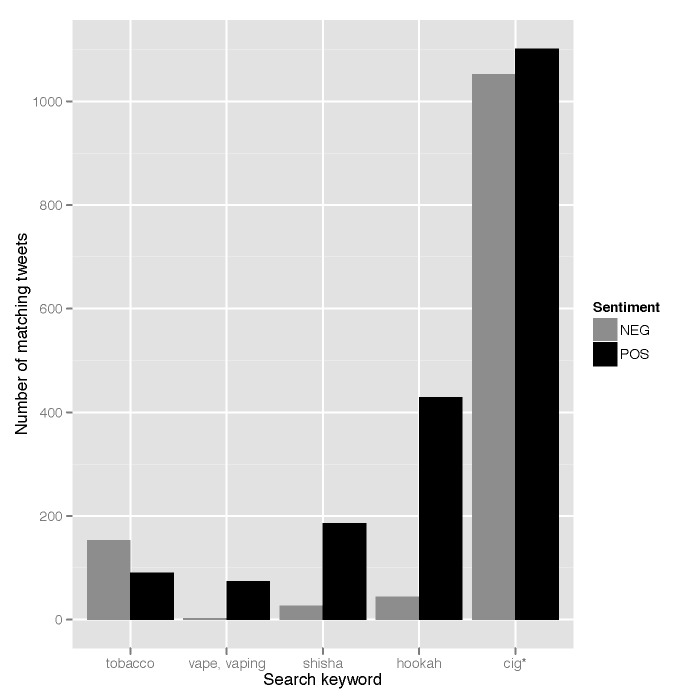
Tweet sentiment by search keyword.

Search keywords associated with each tweet correlated significantly with more general properties, such as sentiment. Examining the five most frequent keywords (representing 96% of tweets), Figure 6 illustrates the tendency for tweets matching the keywords *hookah*, *shisha*, and *vape*/*vaping* to be classified as showing positive sentiment more often than expected by chance, and for those matching *tobacco* to show negative sentiment disproportionately often (note that low frequency keywords—*nicotine*, *waterpipe*, *e-juice,* and *e-liquid—*were excluded). The correlation is highly significant according to a two-tailed chi-square test for independence (χ^2^_4_= 414.50, *P*<.001, Cramer’s *V*=0.36). In this way, a general split in sentiment is observed between, on one hand, the new public health challenges represented by hookah and e-cigarettes, which are viewed more positively, and on the other hand, traditional products such as cigarettes as well as more general references to tobacco, which are viewed more negatively. In other words, smoking hookah is viewed more favorably than smoking traditional tobacco products, even though smoking hookah typically involves smoking tobacco.

### Intercategory Correlations

Correlations between all pairwise combinations of categories in the classification scheme, computed as described in the Methods section, are reported in Figure 1. The highest intercategory correlations were observed between (1) underage usage and social image (0.6), (2) e-cigarettes and marketing (0.54), and (3) positive sentiment and first-person experience (0.47).

### Machine Learning

**Figure 7:**
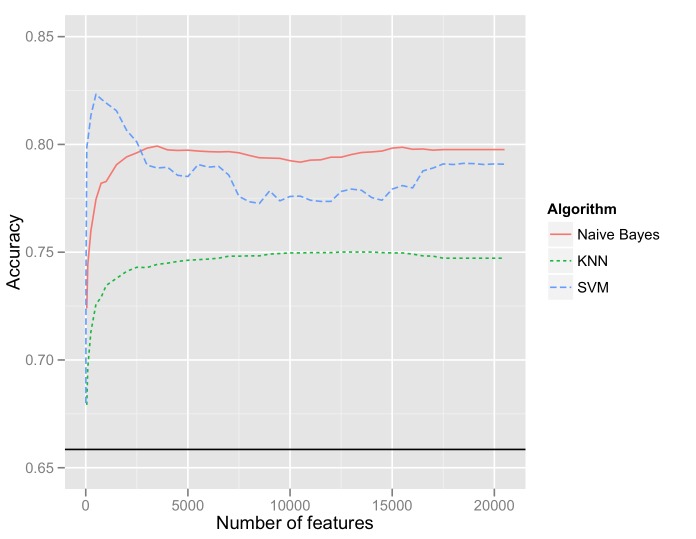
Classification accuracy as a function of number of unigram features for 3 algorithms in the tobacco-relevance task.

The three classification tasks investigated here are (1) relevance to tobacco, (2) positive sentiment toward tobacco, and (3) negative sentiment toward tobacco. For each task, we varied n-gram size, number of features, and machine learning algorithm. In all tasks, unigram feature sets yielded consistently better performance than bigrams on all measures except recall, and bigrams similarly generally outperformed trigrams. A relatively small feature set was generally optimal: Figure 7 illustrates that for the tobacco-relevance task, classification accuracy peaks or levels off with well under 5000 features, and indeed maximum classification accuracy is achieved by a classifier trained on 500 features. SVMs generally yielded the best performance, followed by Naïve Bayes and KNN algorithms, respectively. In discriminating between tobacco-related and -unrelated tweets in order to improve the signal-to-noise ratio in Twitter data, a substantial improvement (82% classification accuracy) over the majority-class baseline (57% classification accuracy) was achieved. Table 1 summarizes performance results for each task using 500 features.

The most informative unigram features for each of the three classification tasks, ranked by log odds ratio, are listed in Table 2. Among the most informative features distinguishing tobacco-related from unrelated tweets are relatively predictable, unambiguous words such as *cigarette, hookah*, and *tobacco*. Several other emergent classes of words are apparent: marketing-related words including *buy* and *http* (typically part of sales website URLs); words semantically or pragmatically associated with tobacco usage such as *smell* and *bar*; and conversational words such as *I’m*, *don’t*, and *lol* that are suggestive of personal expression rather than, for example, news or marketing.

Turning to the most informative features for positive and negative sentiment, several Twitter- specific expressions appear. *gt* and *lt* correspond to the greater-than symbol and the less-than symbol, which are, respectively, explicit tokens of positive and negative sentiment. *smh*, an acronym for *shaking my head*, is a general token of disapproval and is among the most informative features for negative sentiment toward tobacco.

A key point of contrast between highly informative positive words and highly informative negative words is evident in the kind of tobacco product to which they refer. Words related to hookah and e-cigarettes are highly predictive of positive sentiment (respectively, *hookah, hose, shisha; electronic*), whereas *cigarettes* and more general terms such as *smoke* and *tobacco* are predictive of negative sentiment. Discussion of this distinction, as well as its relation to the similar result in the interaction of search keywords and sentiment, is taken up in the next section.

The remaining positive and negative unigrams reveal informative semantic groupings. Words related to recreation and social interaction generally predict positive sentiment toward tobacco, and include *bar, tonight*, and *night*. Marketing-related words, such as *buy*, *free*, *coupon*, *checkout, code*, and *win*, are also prevalent in the positive category. Groupings in the negative category include words related to disgust and social image, such as *nasty*, *unattractive*, *people*, and *girls*, where these last two terms most often occurred in tweets disapproving of particular social groups’ use of tobacco. Finally, words predictive of negative sentiment toward tobacco were also related to health, information, and cessation: *health, kill, study, finds, quit*.

**Table 1:** Performance measures for tobacco relevance, positive sentiment, and negative sentiment classification tasks using 500 features (baseline classification accuracies [majority class] are 57% for relevance, 74% for positive sentiment, and 82% for negative sentiment).

Features		Naïve Bayes					KNN					SVM				
		Acc^a^	*F*	Pre^b^	Rec^c^	Spe^d^	Acc	*F*	Pre	Rec	Spe	Acc	*F*	Pre	Rec	Spe
**Relevance**																
	Unigrams	0.77	0.83	0.73	0.95	0.53	0.73	0.78	0.73	0.83	0.59	0.82	0.85	0.82	0.88	0.75
	Bigrams	0.66	0.77	0.63	0.97	0.24	0.65	0.76	0.63	0.97	0.24	0.73	0.75	0.82	0.69	0.79
	Trigrams	0.61	0.74	0.6	0.99	0.1	0.6	0.74	0.59	0.97	0.11	0.61	0.74	0.59	0.99	0.1
**Positive sentiment**																
	Unigrams	0.76	0.5	0.56	0.45	0.87	0.76	0.37	0.58	0.27	0.93	0.75	0.38	0.53	0.3	0.91
	Bigrams	0.77	0.44	0.62	0.34	0.93	0.76	0.42	0.58	0.33	0.92	0.77	0.43	0.61	0.33	0.92
	Trigrams	0.76	0.26	0.62	0.16	0.96	0.76	0.26	0.62	0.17	0.96	0.76	0.27	0.61	0.17	0.96
**Negative sentiment**																
	Unigrams	0.84	0.52	0.57	0.48	0.92	0.72	0.3	0.27	0.33	0.8	0.83	0.39	0.53	0.3	0.94
	Bigrams	0.85	0.35	0.73	0.23	0.98	0.31	0.3	0.18	0.82	0.2	0.84	0.44	0.59	0.35	0.95
	Trigrams	0.84	0.24	0.76	0.14	0.99	0.22	0.3	0.18	0.94	0.07	0.84	0.37	0.66	0.25	0.97

^a^Acc: accuracy.

^b^Pre: precision.

^c^Rec: recall.

^d^Spe: specificity.

**Table 2:** Most discriminating unigram features for tobacco-related, positive sentiment, and negative sentiment categories, ranked by log odds ratio.

Tobacco-related	Positive sentiment	Negative sentiment
cigarette	hookah	lt
hookah	cigar	cigarettes
lt	bar	smell
smoking	tonight	hate
tobacco	gt	smoke
cigs	electronic	people
electronic	night	disgusting
http	good	tobacco
smell	code	finds
cigar	checkout	study
im	love	girls
bar	lol	alcohol
hate	free	nasty
day	ecigarette	unattractive
dont	buy	smh
gt	hose	smells
buy	win	kill
lol	coupon	health
people	flavored	mouth
good	shisha	quit

## Discussion

### Principal Findings

The Twitter surveillance results converge in several key classes of findings, which we discuss in turn in this section. First, the content analysis allows for a general pulse or snapshot to be taken of tobacco-related discussion on Twitter. Second, new insight can be gained into causes for positive and negative sentiment toward tobacco, especially with respect to hookah and e-cigarettes. Finally, several potential opportunities for tobacco education emerge, and we discuss them in the context of future research directions.

The relative prevalence of the various categories in the content analysis reflect a general pulse of tobacco-related discussion on Twitter. By far the most common categories are personal experiences and opinion, affirming the value of Twitter in assessing public sentiment and informedness. The next most common genre, marketing, is followed relatively distantly by information and news, and most tweets in these categories are not posted by recognized health or news organizations. In sum, reliable information is far less accessible on Twitter than are opinions, marketing posts, and information from unverified sources, indicating potential for greater public education in tobacco prevention policies.

Twitter surveillance allows for new insight into the correlates of positive and negative sentiment toward tobacco. Among Twitter users that post about tobacco in our dataset, sentiment is overall more positive than negative, even with marketing posts excluded. The strongest correlate of positive sentiment is first-hand personal experience, while negative sentiment correlates more strongly with opinion. In this regard, Twitter surveillance may reveal insights not available through surveys, where participants do not spontaneously relate experiences to an audience of friends and followers and are instead more likely to express more carefully crafted opinions. Indeed, surveys may thus underestimate the prevalence of positive sentiment toward tobacco.

Among the clearest correlates of positive sentiment are hookah and e-cigarettes. On all measures computed in this study, including (1) correlations between categories in the annotation scheme, (2) correlations between search keywords and sentiment, and (3) most discriminating unigram features for positive and negative sentiment, a split emerged between, on one hand, hookah and e-cigarettes as corresponding to positive sentiment, and on the other hand, other products as well as general references to tobacco corresponding to negative sentiment. Especially in the case of hookah, such a split may indicate a disconnect in public perception between popular tobacco products and risk factors associated with tobacco use in general, presenting a distinct opportunity for outreach and education by tobacco control organizations.

Social relationships, especially among younger users, emerge as another key component of positive sentiment toward tobacco on Twitter, often in conjunction with products such as hookah. In the following example, tobacco usage is a central component of a positive experience in a social relationship: “Smoking that good hookah with the bro Sultan! #GoodOldDays #brotherforlife”. These positive tobacco-centric social experiences also frequently involve young or under-age users: “Beer ponggg / hookah round 2 with my goons waddduppppppp. I love when my parents rnt home!”

In a related vein, these products are also associated with initiation of tobacco usage, as in the following: “an e-cigarette salesman at a mall to Parris and I: ‘Do you guys smoke?’ ‘No.’ ‘Do you wanna start?’. ”

In this way, positive sentiment toward tobacco appears to participate in a complex interaction between newer products such as hookah and e-cigarettes, younger users, and positive social experiences.

A social component is also central to negative sentiment toward tobacco. Categories corresponding to disgust and stereotypes were among the most highly correlated with negative sentiment, in fact outranking the explicit health category. A key distinction, however, is that while the category of social image correlated with negative sentiment, social relationships correlated with positive sentiment. Taken together, these findings indicate that social factors are central in driving sentiment toward tobacco and suggest that public health campaigns may do well to make use of this correlation.

Several novel findings, in sum, speak to the unique insights available through Twitter surveillance. All measures converged on an emergent distinction between two recently popular tobacco products, hookah and e-cigarettes, which corresponded to positive sentiment, and other products as well as references to tobacco more generally, which corresponded to negative sentiment. Sentiment toward tobacco overall among Twitter users is more positive than negative, affirming Twitter’s value as a resource to understand positive sentiment in developing improved prevention policies. Negative sentiment is equally useful; for example, observed high correlations between negative sentiment and social image, but not health issues, may usefully inform tobacco control strategies. Twitter surveillance further reveals opportunities for education. Positive sentiment toward the term *hookah* but negative sentiment toward *tobacco* suggests a disconnect in users’ perceptions of the health effects of hookah (ie, hookah is not regarded in the same negative light as traditional tobacco products). Finally, machine classification of tobacco-related posts shows a promising edge over strictly keyword-based approaches, yielding an improved signal-to-noise ratio and paving the way for automated tobacco surveillance applications.

### Limitations

The work reported in this paper does have some limitations. First, we harvested our data from the free 1% Twitter feed, rather than the full Twitter firehose. Second, our annotated dataset was relatively small, and there is some risk of our model overfitting. Third, the number of smoking keywords used to identify tobacco-relevant tweets was quite limited. It would be useful to augment our keyword list with tobacco-related slang (eg, “cancer sticks”, “coffin nails”) or electronic cigarette brands (eg, “blucigs”, “greensmoke”). Fourth, in this work we have concentrated exclusively on analyzing tobacco-related tweets using natural language processing rather than on the social network aspect of Twitter (ie, we did not analyze the characteristics of those tweets most likely to be retweeted). Finally, one key issue that we have not addressed in this work is the role of novelty effects in attitudes towards e-cigarettes (ie, will interest in the products be sustained over time?). In future work we will address all these issues.

Our medium-term goal, building on the work described in this paper, is to create a Web-based social media monitoring system for tobacco-related products and smoking behaviors, integrating natural language processing, geographical information systems, and social network analysis to provide a service that will allow public health workers and other interested parties to monitor and track public attitudes towards a range of both established and emerging tobacco products, and to formulate policy and interventions accordingly.

## Multimedia Appendix 1

Annotation scheme.

## Multimedia Appendix 2

Evaluation metrics.

## Abbreviations

API: Application Programming Interface
CDC: Centers for Disease Control
KNN: k-nearest neighbors
SVM: support vector machine
USFDA: United States Federal Drug Administration

## References

[1] Eysenbach G (2009). Infodemiology and infoveillance: framework for an emerging set of public health informatics methods to analyze search, communication and publication behavior on the Internet. J Med Internet Res.

[2] Twitter turns six.

[3] Mislove A, Lehmann S, Ahn Y, Onnela J, Rosenquist J (2011). Understanding the demographics of Twitter users. Proceedings of the Fifth International AAAI Conference on Weblogs and Social Media.

[4] Smith A, Brenner J Twitter use 2012.

[5] Doan S, Vo B, Collier N (2011). An analysis of Twitter messages in the 2011 Tohoku Earthquake.

[6] Collier N, Son NT, Nguyen NM (2011). OMG U got flu? Analysis of shared health messages for bio-surveillance. J Biomed Semantics.

[7] Chew C, Eysenbach G (2010). Pandemics in the age of Twitter: content analysis of Tweets during the 2009 H1N1 outbreak. PLoS One.

[8] Krieck M, Dreesman J, Otrusina L, Denecke K (2011). A new age of public health: identifying disease outbreaks by analyzing tweets. Proceedings of Health WebScience Workshop, ACM Web Science Conference.

[9] Paul M, Dredze M (2011). You are what you tweet: analyzing Twitter for public health. Proceedings of the 5th International AAAI Conference on Weblogs and Social Media.

[10] Culotta A (2010). Towards detecting influenza epidemics by analyzing Twitter messages. Proceedings of the First Workshop on Social Media Analytics.

[11] Scanfeld D, Scanfeld V, Larson EL (2010). Dissemination of health information through social networks: twitter and antibiotics. Am J Infect Control.

[12] Heaivilin N, Gerbert B, Page JE, Gibbs JL (2011). Public health surveillance of dental pain via Twitter. J Dent Res.

[13] West J (2012). Temporal variability of problem drinking on Twitter. Open Journal of Preventive Medicine.

[14] Golder SA, Macy MW (2011). Diurnal and seasonal mood vary with work, sleep, and daylength across diverse cultures. Science.

[15] World Health Organization (2011). WHO Report on the global tobacco epidemic 2011: warning about the dangers of tobacco.

[16] Armour B, Woolery T, Malarcher A, Pechacek T, Husten C (2005). Annual smoking- attributable mortality, years of potential life lost, and productivity losses. Morbidity and Mortality Weekly Report.

[17] US Department of Health and Human Services (2004). The health consequences of smoking: a report for the Surgeon General.

[18] Freeman B (2012). New media and tobacco control. Tob Control.

[19] Lowe JB, Barnes M, Teo C, Sutherns S (2012). Investigating the use of social media to help women from going back to smoking post-partum. Aust N Z J Public Health.

[20] Prochaska JJ, Pechmann C, Kim R, Leonhardt JM (2012). Twitter=quitter? An analysis of Twitter quit smoking social networks. Tob Control.

[21] Prier K, Smith M, Giraud-Carrier C, Hanson C (2011). Identifying health related topics on Twitter. Social Computing, Behavioral-Cultural Modeling and Prediction.

[22] U.S. Food and Drug Administration RFA.

[23] Blei D, Ng A, Jordan M (2003). Latent Dirichlet allocation. J Mach Learn Res.

[24] Neuendorf K (2001). The Content Analysis Guidebook.

[25] Rogers JM (2008). Tobacco and pregnancy: overview of exposures and effects. Birth Defects Res C Embryo Today.

[26] Cobb C, Ward KD, Maziak W, Shihadeh AL, Eissenberg T (2010). Waterpipe tobacco smoking: an emerging health crisis in the United States. Am J Health Behav.

[27] Smith JR, Edland SD, Novotny TE, Hofstetter CR, White MM, Lindsay SP, Al-Delaimy WK (2011). Increasing hookah use in California. Am J Public Health.

[28] Grekin ER, Ayna D (2012). Waterpipe smoking among college students in the United States: a review of the literature. J Am Coll Health.

[29] Cobb CO, Shihadeh A, Weaver MF, Eissenberg T (2011). Waterpipe tobacco smoking and cigarette smoking: a direct comparison of toxicant exposure and subjective effects. Nicotine Tob Res.

[30] Smith SY, Curbow B, Stillman FA (2007). Harm perception of nicotine products in college freshmen. Nicotine Tob Res.

[31] Primack BA, Hopkins M, Hallett C, Carroll MV, Zeller M, Dachille K, Kim KH, Fine MJ, Donohue JM (2012). US health policy related to hookah tobacco smoking. Am J Public Health.

[32] Rice VH (2012). Water pipe smoking among the young: the rebirth of an old tradition. Nurs Clin North Am.

[33] Foulds J, Veldheer S, Berg A (2011). Electronic cigarettes (e-cigs): views of aficionados and clinical/public health perspectives. Int J Clin Pract.

[34] Choi K, Fabian L, Mottey N, Corbett A, Forster J (2012). Young adults' favorable perceptions of snus, dissolvable tobacco products, and electronic cigarettes: findings from a focus group study. Am J Public Health.

[35] Freiberg M (2012). Options for state and local governments to regulate non-cigarette tobacco products. Ann Health Law.

[36] Ayers JW, Ribisl KM, Brownstein JS (2011). Tracking the rise in popularity of electronic nicotine delivery systems (electronic cigarettes) using search query surveillance. Am J Prev Med.

[37] Hastie T, Tibshirani R, Friedman J (2009). The Elements of Statistical Learning: Data Mining, Inference, and Prediction, Second Edition.

[38] Yang Y, Pedersen J (1997). A comparative study on feature selection in text categorization. Proceedings of the Fourteenth Annual Conference on Machine Learning.

[39] Forman G (2003). An extensive empirical study of feature selection metrics for text classification. The Journal of Machine Learning Research.

[40] Rainbow Toolkit.

[41] Porter Stemmer.

[42] SMART stoplist: list/english.

