# Supplementary material for: Using Twitter to Examine Smoking Behavior and Perceptions of Emerging Tobacco Products
Source: J Med Internet Res. 2013 Aug 29;15(8):e174. doi: 10.2196/jmir.2534 (PMC3758063; doi:10.2196/jmir.2534)
Supplement: Supplementary file 1 [file jmir_v15i8e174_app1.pdf]

Appendix 1 – Annotation Scheme

**GENRE** (assign each tweet to zero or more of these categories)		
**Experience** (first-hand)	Reports a personal use of tobacco	*last week we smoked cigars and pretented to be dragons looool I love big bang theory*
**Experience** (other)	Reports someone else's use of tobacco.	*That awkward moment when you see a six year old holding an unlit cigarette about to light that shit. #sadface*
**Activism**	Alarm or call for change in tobacco policy	*This July 4th, celebrate your freedom from @FDATobacco nazis by smoking a fine cigar. (They're trying hard to remove this freedom, folks.)*
**Cultural reference**	Song lyric, movie title, etc	*smoked one too many cigarettes, heard one too many lies*
**Humor**	Formulaic joke, bumper sticker, etc.	*Pass with care. I chew tobacco.*
**News**	News item	*U.S. tobacco crop up despite dwindling industry http://t.co/QPBlpYl*
**Info/resource**	Factoid or informational resource	*Hazzards of smokeless cigarettes http://t.co/FR47HiV*
**Marketing**	Sale of tobacco product/accessory	*Try our fantastic Tangerine flavored ecigarette eliquid! Get 20% off at checkout with coupon code - twitter http://t.co/QeCZ3D3*
**Opinion**	Personal opinion related to tobacco.	*@Mahonopoly smoke cigarettes, it only sucks when you die #YOLO*

**THEME** (assign each tweet to zero or more of the lowest level categories below)		
Risk, starting, & stopping: *tweets broadly based on whether people should use tobacco*		
**Cessation**	Quit-smoking resources, personal cessation progress, etc	*i needa quit smoking cigarettes dead ass*
**Starting**	How people started tobacco usage, desires to start, etc.	*Em just offered me a fag and I said yes. Am I a smoker now? #peerpressure naughtyyy*
**Health**	Direct or indirect reference to health consequences of tobacco usage	*Electronic health cigarette http://t.co/sXyLpvf2*
**Downplayed risk**	Downplayed risk or rationalization of tobacco usage	*cancer is not just caused by cigarettes, but every choice you make in life and then a hint of bad luck*
**The new tobacco**	Activities that are claimed to have taken tobacco's place as addictive or dangerous cultural phenomena, or general reference to tobacco as symbolic of addiction	*Facebook is where cigarettes used to be: popular, addictive, and with recognized downsides that most people accept*
Product: *tweets centered on tobacco products themselves*		
**Commodity**	Tobacco products as an item of value; cost; obtaining tobacco products.	*#Budget2012 single parents, time to decide, quit smoking or dont feed your kids*
**Connoisseurship**	Details of product quality and discrimination; may include brand names	*I'm so god damn tired of getting disgusting menthol cigarettes when I ask someone for one.*
**E-cigarettes**	Reference to electronic cigarettes	*Jan. 2010 e-cigarette reviews http://t.co/am287dRQ*
**Hookah**	Reference to hookah	*#UknowUasianWhen you go to someones house just to do shisha and take pictures with smoke coming out your mouth like a nob.*
Sociocultural factors: *role of tobacco in social image and relationships*		
**Image & stereotypes**	social image, user stereotypes, and other social indexicality associated with tobacco	*Hood bitches get back shots and smoke black & milds *regular flavor* @ the same time*
**Social relationships**	Effects on family and other social relationships	*Sitting in front of a random hotel having sister bonding time while smoking cigars.*

continued on next page

continued from previous page

**THEME** (assign each tweet to zero or more of the lowest level categories below)		
Consumption: *details of actual tobacco usage*		
**Pleasure**	Tobacco usage as a pleasurable activity	*If I was Steve Jobs, I could definitely use a cigarette after the massive blowjob Twitter gave him today.*
**Craving**	Possibly related to stress relief	*omg, i havent had a cigarette in a good 3 days, this is going to be a bad week. fuck me.*
**Disgust**	Tobacco users or usage as repulsive	*Ughhhh ! I smell like cigarettes. I also have randm ass texts from last night. Idek.*
**Underage usage**	Tobacco usage by minors	*Beer ponggg / hookah round 2 with my goons wadddupppppp. I love when my parents rnt home!*
**Other substances**	Tobacco in association with other addictive substances such as alcohol, caffeine, or marijuana	*cigarettes are only good when im drunk*
**Backgrounded**	Everyday or non-foregrounded references to tobacco usage.	*Goddamn.. These winds are crazy. Almost blew away just smoking a cigarette.*
Industry & politics: *tweets concerning tobacco industry, regulation, economy, taxation*		
**Industry**	Tobacco industry-related	*TIL In 2009, the company Reynolds American (the second-largest tobacco company in the US) closed a deal for $44.5... http://t.co/RQ9subQI*
**Politics**	Political aspects including regulation and smoking bans	*I would like to thank those involved in writing the budget when it comes to the increase in cigarettes! #filthyhabit #shouldbemorethough*

**SENTIMENT** (assign each tweet to positive, negative, or neither)		
**Positive**	Positive sentiment toward tobacco	*Smoking that good hookah with the bro Sultan! #GoodOldDays #brotherforlife*
**Negative**	Negative sentiment toward tobacco	*omg i hate the smell of ciggeretts*
**[Neither]**	Neutral reference to tobacco	*tinarosa7 btw i dont knw why they smoke shisha nbut i knw that its not allowd in islam*
