# Supplementary material for: Using Twitter to Examine Smoking Behavior and Perceptions of Emerging Tobacco Products
Source: J Med Internet Res. 2013 Aug 29;15(8):e174. doi: 10.2196/jmir.2534 (PMC3758063; doi:10.2196/jmir.2534)
Supplement: Supplementary file 2 [file jmir_v15i8e174_app2.pdf]

Appendix 2 – Evaluation Metrics

Evaluation metrics used in this study (precision, recall, specificity, and F–score) are defined below with respect to Table A1:

$$\text{Accuracy} = \frac{a+d}{a+b+c+d}$$

$$\text{Precision} = \frac{a}{a+b}$$

$$\text{Recall} = \frac{a}{a+c}$$

$$\text{Specificity} = \frac{d}{b+d}$$

$$F\text{-score} = \frac{2 \times \text{Precision} \times \text{Recall}}{\text{Precision} + \text{Recall}}$$

	**REL correct**	**Non-REL**
Assigned REL	a	b
Assigned non-	c	d

Table A1: Contingency table for calculating classification accuracy (REL is "Relevant" and non-REL is "Non-Relevant"
